# SERPINA5 promotes tumour cell proliferation by modulating the PI3K/AKT/mTOR signalling pathway in gastric cancer

**DOI:** 10.1111/jcmm.17514

**Published:** 2022-08-24

**Authors:** Meiyang Fan, Xiaofan Xiong, Lin Han, Lingyu Zhang, Shanfeng Gao, Liying Liu, Xiaofei Wang, Chen Huang, Dongdong Tong, Juan Yang, Lingyu Zhao, Yuan Shao

**Affiliations:** ^1^ Department of Otolaryngology & Head Neck The First Affiliated Hospital of Xi'an Jiaotong University Xi'an China; ^2^ Department of Cell Biology and Genetics, School of Basic Medical Sciences Xi'an Jiaotong University Health Science Center Xi'an China; ^3^ Key Laboratory of Environment and Genes Related to Diseases (Xi'an Jiaotong University) Ministry of Education of China Xi'an China; ^4^ Department of Tumor and Immunology in precision medicine institute Western China Science and Technology Innovation Port Xi'an China

**Keywords:** cell proliferation, gastric cancer, SERPINA5, signal regulation, tumour therapy

## Abstract

SERPINA5 belongs to the serine protease inhibitor superfamily and has been reported to be lowly expressed in a variety of malignancies. However, few report of SERPINA5 in gastric cancer has been found. The purpose of this study was to determine the role of SERPINA5 in GC and to investigate potential tumorigenic mechanisms. We performed qPCR to determine the level of SERPINA5 expression in GC. We used public databases to evaluate whether SERPINA5 could be utilized to predict overall survival and disease‐free survival in GC patients. We also knocked down the expression of SERPINA5 and evaluated its effect on cell proliferation and migration. Furthermore, we explored the signal pathways and regulatory mechanisms related to SERPINA5 functions. According to our findings, SERPINA5 was shown to exhibit high expression in GC. Notably, SERPINA5 was prognostic in GC with high expression being unfavourable. SERPINA5 was further observed to promote GC tumorigenesis by modulating GC cell proliferation ability. Mechanically, SERPINA5 could inhibit CBL to regulate the PI3K/AKT/mTOR signalling pathway, thereby promoting GC carcinogenesis progression. These results highlight the important role of SERPINA5 in GC cell proliferation and suggest that SERPINA5 could be a novel target for GC treatment and a predictor for GC prognosis.

## 1 INTRODUCTION

Gastric cancer (GC) remains one of the most frequent cancers worldwide.^1^ Several genetic mechanisms, such as oncogene activation or tumour suppressor gene inactivation, are essential factors in the tumorigenesis of GC.^2^ For example, loss of p53^3^ and AKT^4^ have been linked to the development of GC. Surgical removal, which is only possible in 25–30 per cent of patients, has been the only way to cure GC until recently.^5^ Despite this, GC treatment still remains challenging due to the disease's complexity and difficulty in detecting it early on.^6^ Hence, understanding the events that drive GC progression and development is of paramount importance for identifying effective therapeutic targets.

SERPINA5 (also known as Protein C Inhibitor, PCI) is a serine protease inhibitor that can physiologically inhibit activated protein C (APC), a major anticoagulant protease.^7^, ^8^ SERPINA5 was found in a variety of human tissues and first isolated from human plasma, with most of SERPINA5 generated in the liver,^9^ but also synthesized in the kidneys.^10^ Previous reports showed that SERPINA5 exhibited low expression in a range of cancers, including renal, breast, prostate and ovarian tumours.^11^, ^12^, ^13^, ^14^ SERPINA5 has recently been proposed as a novel susceptibility locus for papillary thyroid cancer, with SERPINA5 having a significantly prognostic value in colorectal cancer.^15^, ^16^ SERPINA5 plays important roles in tumours, including preventing metastasis and anti‐angiogenesis, since it engages in a wide range of biological activities, including inflammation.^17^, ^18^ In additional, researchers demonstrated that host SERPINA5 inhibit tumour growth, but promote tumour metastasis in B16 melanoma.^19^ In GC, a study of survival prediction model showed that SERPINA5 is considered as one of the GC progression‐related genes.^20^ Nevertheless, the role of SERPINA5 in GC is largely unknow and needs to be fully studied.

Casitas B‐lineage lymphoma (CBL) containing an N‐terminal tyrosine kinase–binding (TKB) domain, RING domain, C‐terminal phosphorylation sites and a ubiquitin‐association domain has an E3 ubiquitin ligase activity and signalling adaptor function,^21^ which plays a key role in basic biological functions such as cell invasion and proliferation. CBL mutations in patients with haematological malignancies had previously been the focus of research.^22^, ^23^, ^24^ According to the recent emerging evidence, CBL appears to have a significant role in human solid organ cancers.^25^, ^26^ CBL's capacity to control tumour growth inhibitory effects makes it a promising target for cancer therapy.

In this research, our findings suggested that SERPINA5 exhibited high expression with unfavourable prognosis in GC. Ectopic expression of SERPINA5 could promote GC cell proliferation ability. Mechanically, SERPINA5 could interact with CBL and they were negatively correlated. SERPINA5 could inhibit CBL to modulate the PI3K/AKT/mTOR signalling pathway, thereby promoting GC carcinogenesis progression. Taken together, our results not only highlight the important function of SERPINA5 in regulating the proliferation potential of GC cells, but also indicate that SERPINA5 could be a novel target for GC treatment and an indicator for GC prognosis.

## 2 METHODS AND MATERIALS

### 2.1 Human cell lines and cell culture

The GC tumour tissues and paired normal tissues were taken from patients who underwent surgical removal at the First Affiliated Hospital of Xi'an Jiaotong University. The Biomedical Ethics Committee of the Medical Department of Xi'an Jiaotong University gave their approve to this work. All participants involved in this study gave their informed consent.

All cell lines used (GES‐1, MKN‐28, BGC‐823, SGC‐7901, MKN‐45 and AGS) in this study were provided from Cell Bank (Genechem,). Before doing any research, the Cell Bank validated all of the cell lines and screened them for mycoplasma. Cells were incubated at 37°C with 5% CO2 using RPMI 1640 medium (Thermo Fisher Scientific, Inc.) added with 10% foetal bovine serum (Biological Industries).

### 2.2 Cellular transfection

GenePharma created specific small interfering (si) RNAs. As a control, nonspecific siRNA sequences were utilized (si‐CON). Lipofectamine 2000 (Thermo Fisher Scientific, Inc.) was utilized as a transfection reagent, based on provided directions. For sequence details regarding all oligonucleotides, see Table 1.

**TABLE 1:** RNA oligonucleotides used in this work

Name	Sequences	
	Sense (5′–3′)	Antisense (5′–3′)
si‐SERPINA5‐1	GGCACCCAAGAGCAAGACUTT	AGUCUUGCUCUUGGGUGCCTT
si‐SERPINA5‐2	CGCUGAGGAAGUGGCUUAATT	UUAAGCCACUUCCUCAGCGTT
si‐CBL‐1	GCUCGGCUCCAGAAAUUCATT	UGAAUUUCUGGAGCCGAGCTT
si‐CBL‐2	CCGGCACUCACUUCCAUUUTT	AAAUGGAAGUGAGUGCCGGTT
si‐CON	UUCUCCGAACGUGUCACGUTT	ACGUGACACGUUCGGAGAATT

### 2.3 Quantitative real‐time polymerase chain reaction (qRT‐PCR)

Total RNA was extracted using TRIzol® reagent (Invitrogen), followed by reversing transcription using a PrimeScript™ RT Kit (Takara Bio, Inc.). qRT‐PCR were then performed using an IQ5 instrument (Bio‐Rad Laboratories, Inc.) together with SYBR Green Ex Taq™ II (Takara Bio, Inc.). We analysed relative gene expression using the 2^−ΔΔCt^ method,^27^ using GAPDH as a control. The programs for qRT‐PCR were as follows: 10 min at 95°C; 40 cycles of 95°C for 15 s, 60°C for 60 s and 72°C for 30 s. For the primer sequences used for qRT‐PCR, see Table 2.

**TABLE 2:** Primers used in this work

Gene name	Primer sequences	
	Forward (5′–3′)	Reverse (5′–3′)
SERPINA5	AGCAATGCGGTCGTGAT	TCCGGTCCAGGAGGTAG
CBL	TAGGCGAAACCTAACCAAACTG	AGAGTCCACTTGGAAAGATTCCT
GAPDH	TGAAGGTCGGAGTCAACGGATT	CCTGGAAGATGGTGATGGGATT

### 2.4 MTT assay

We evaluated the effect of SERPINA5 on GC cell proliferation by performing 3‐(4, 5‐dimethylthiazol‐2‐yl)‐2, 5‐diphenyl‐tetrazolium bromide (MTT) experiment. A total of 3500 cells were planted to the 96‐well plates and transfected the following day. After 24, 48 and 72 h, 10 μl MTT (5 mg/mL) was supplied per well and then keep incubating at 37°C. Four hours later, all liquid was removed, with 150 μl of Dimethyl Sulfoxide (DMSO) added per well. A plate reader was used to record the absorbance at 490 nm.

### 2.5 Colony formation assay

After being transfected for 24 h, 2000 cells were planted into 12‐well plates. After that, the cells were cultured for 1–2 weeks, followed by fixation using 4% paraformaldehyde, crystal violet solution (Sigma Aldrich; Merck KGaA) staining, imaging and quantification with the Quantity One® program (Bio‐Rad Laboratories, Inc.).

### 2.6 Cell apoptosis analysis

Cell apoptosis assay was assessed using an Annexin‐V Apoptosis Detection Kit (Life Technologies,) to determine whether SERPINA5 affect apoptosis of GC cells. MKN‐28 and BGC‐823 cells were plated at 2 × 10^5^ per well in six‐well plates and transfected with SERPINA5 siRNA and control siRNA. At 48 h post‐transfection, cells were collected and washed with PBS together with binding buffer. AnnexinV–FITC and PI were then used for intracellular staining. The apoptosis cells as a percentage were analysed using a flow cytometer (Becton‐Dickinson,).

### 2.7 Cell cycle analysis

Cell cycle analysis was performed to evaluate the proportion of GC cells during the cell cycle progress. In six‐well plates, 2 × 10^5^ MKN‐28 and BGC‐823 cells were planted. After a 24 h incubation period, cells were cultured using serum‐free medium for another 24 h period before transfected with SERPINA5 siRNA and control siRNA. After an additional 24 h, the cells were collected, rinsed with cold PBS and fixed in 70% ethanol at 4°C overnight. Following that, the cells were washed 3 times using PBS and then stained using RNase A (0.1 mg/mL) and Propidium Iodide (0.05 mg/mL). A flow cytometry (Becton‐Dickinson, USA) was then used for cell cycle analysis.

### 2.8 Cell migration assay

The effect of SERPINA5 on GC cell migration was investigated using a wound healing experiment. As growing cells to confluence in 6‐well plates, a sterile pipette tip was used to create a scratch wood. The closure of this wood was then measured at 24, 48 and 72 h via imaging.

### 2.9 Western blot analysis

The total protein from GC cells were extracted using radioimmune precipitation (RIPA) buffer (Wolsen Biotech,) supplemented with phosphatase and protease inhibitors as well as phenylmethyl sulfonyl (PMSF) (Sigma). A BCA assay kit (Takara Bio, Inc.) was then used for quantifying protein concentration. After that, prepared samples were isolated via SDS‐PAGE and transferred to polyvinylidene difluoride (PVDF) membranes (Millipore, CA). Ten per cent skim milk was then used for blocking for 90 min, followed by incubating overnight at 4°C using appropriate primary antibodies. The membranes were treated with HRP‐linked secondary antibodies for 1.5 h on the second day (Cell Signalling Technology, Inc.). The membranes were cleaned 3 times with Tris‐buffered saline‐Tween 20 after each incubation step. At the end, the membranes were observed using an enhanced chemiluminescence detection system (Syngene Europe) and analysed with ImageJ software (National Institutes of Health). For primary antibodies used in this study, see Table 3.

**TABLE 3:** Primary antibodies used in this work

Antibody	Product number	Brand	Working dilutions
anti‐SERPINA5	10,673‐1‐AP	Proteintech	1:500
anti‐CBL	25,818‐1‐AP	Proteintech	1:2000
anti‐Akt	#9272	Cell Signal Technology	1:1000
anti‐phospho‐Akt	#4060	Cell Signal Technology	1:2000
anti‐mTOR	#2983	Cell Signal Technology	1:1000
anti‐phospho‐mTOR	#5536	Cell Signal Technology	1:1000
anti‐CyclinD1	#60186‐1‐Ig	Proteintech	1:1000
anti‐CDK4	#11026‐1‐AP	Proteintech	1:1000
anti‐Bax	#50599‐2‐Ig	Proteintech	1:2000
anti‐Bcl‐2	#15071	Cell Signal Technology	1:1000
anti‐Caspase‐3	#19677‐1‐AP	Proteintech	1:1000
anti‐PARP	#9532	Cell Signal Technology	1:1000
Anti‐β‐Actin	#AB0035	Abways Technology	1:5000

### 2.10 Co‐immunoprecipitation

Extracted proteins from MKN‐28 cells were mixed together with the primary antibody, which was slowly shaken at 4°C overnight. The DynabeadsTM Protein G (Invitrogen) were washed twice in PBS before being used to prepare a 50% protein G beads working solution that was then added into the abovementioned antigen–antibody mixture and slowly shaken at 4°C for 4 h. By magnetic separation, the clear liquid was discarded, and the protein G beads were harvested and washed, and the samples were separated by boiling for next experiment.

### 2.11 Statistical analysis

For graphs preparation and data analysis, GraphPad Prism 7 software (GraphPad Software, Inc.) was used. All results were displayed as mean ± standard deviation and analysed via Student's *t*‐test. SPSS v22.0 (SPSS, Inc.) was used to perform certain data analysis. The significance level was set at *p* < 0.05.

## 3 RESULTS

### 3.1 Up‐Regulation of SERPINA5 is correlated with poor prognosis in GC

**FIGURE 1:**
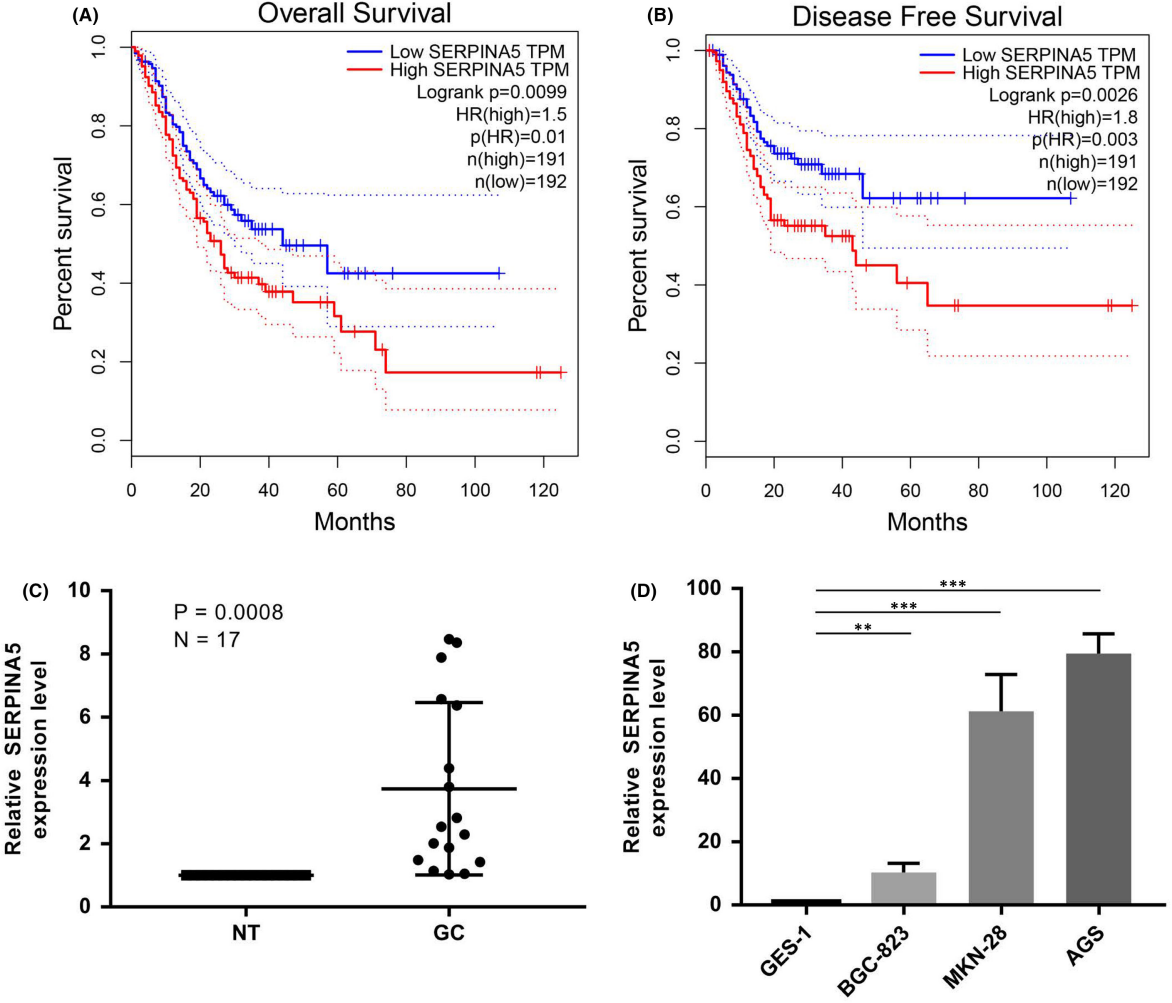
Up‐regulation of SERPINA5 is correlated with poor prognosis in GC. Kaplan–Meier survival curves showing the effect of SERPINA5 expression on (A) overall survival rates and (B) disease‐free survival rate in patients with GC. (C): SERPINA5 expression in 17 paired human gastric cancer and adjacent normal tissues. (D): Expression of SERPINA5 in normal GES‐1 cells and three gastric cancer cell lines. *p < 0.05, **p < 0.01, ***p < 0.001

To investigate whether SERPINA5 was a potential oncogene in GC, we first analysed from the Gene Expression Profiling Interactive Analysis (GEPIA) database to determine whether SERPINA5 is related to the overall survival (OS) and disease‐free survival (DFS) of GC patients. According to the Kaplan–Meier curves, high expression of SERPINA5 is consistently correlated with shorter OS and DFS, suggesting a prognostic value of SERPINA5 in GC and may serve as an independent predictor (Figure 1A,B). We then examined the expression levels of SERPINA5 in GC tissues and corresponding non‐tumour tissues via qRT‐PCR. SERPINA5 expression was much higher in tumour samples than in corresponding normal tissues, as shown in Figure 1C. Moreover, when compared to the normal gastric epithelial cell line (GES‐1), SERPINA5 was also detected to be considerably elevated in 3 human GC cell lines (BGC‐823, MKN‐28 and AGS) (Figure 1D). Taken together, our results suggested that increased SERPINA5 expression is correlated with poor prognosis in GC.

### 3.2 SERPINA5 knockdown inhibits GC cell viability and proliferation

**FIGURE 2:**
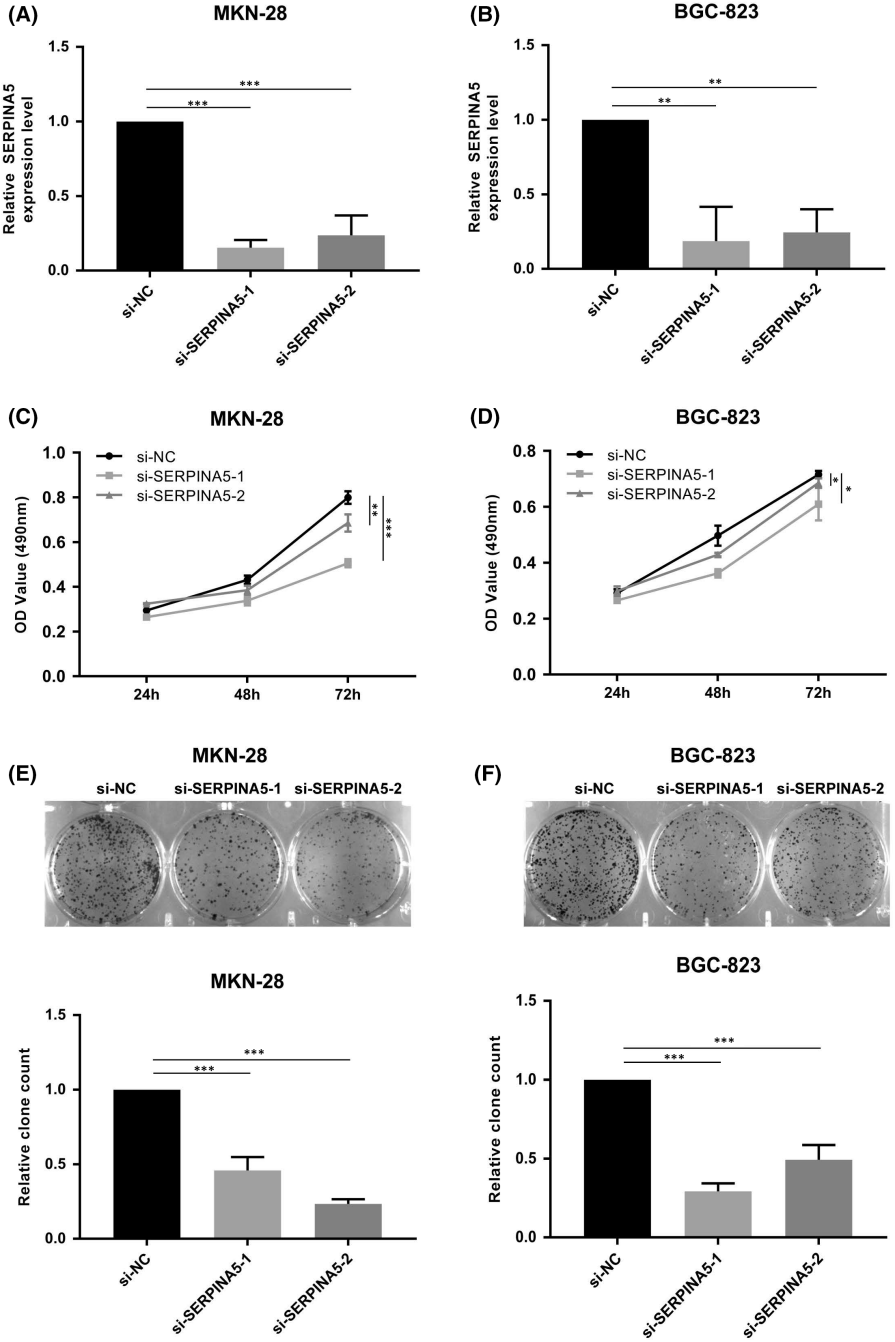
SERPINA5 knockdown inhibits cell viability and proliferation. Expression of SERPINA5 in si‐CON and si‐SERPINA5 transfected (A) MKN‐28 and (B) BGC‐823 cells. MTT assay showing the viability of (C) MKN‐28 and (D) BGC‐823 cells transfected with si‐CON or si‐SERPINA5. Representative results of colony formation in (E) MKN‐28 and (F) BGC‐823 cells, respectively. *p < 0.05, **p < 0.01, ***p < 0.001

We next knocked it down using siRNAs that target human SERPINA5 in GC cell lines to further explore the oncogenic role of SERPINA5 in GC. Firstly, expression levels of SERPINA5 were detected following transfection, the results showed that SERPINA5 were successfully knocked down in MKN‐28 and BGC‐823 cells (Figure 2A,B). Next, cell proliferation ability was evaluated by MTT and colony formation assays. As shown in Figure 2C,D, MKN‐28 and BGC‐823 cells grew less efficiently following SERPINA5 knockdown relative to control cells. Likewise, As shown in the results, silencing of SERPINA5 induced a decrease in colony formation number and size for both tested cell lines (Figure 2E,F), consistent with the MTT assay findings and showing that SERPINA5 can significantly promotes GC cells viability and proliferation. To assess whether SERPINA5 influenced GC cell migration, we also conducted wound healing assays, but the results showed no significant difference (Figure S1).

### 3.3 SERPINA5 knockdown induces G0/G1 phase arrest and promotes apoptosis in GC cell lines

**FIGURE 3:**
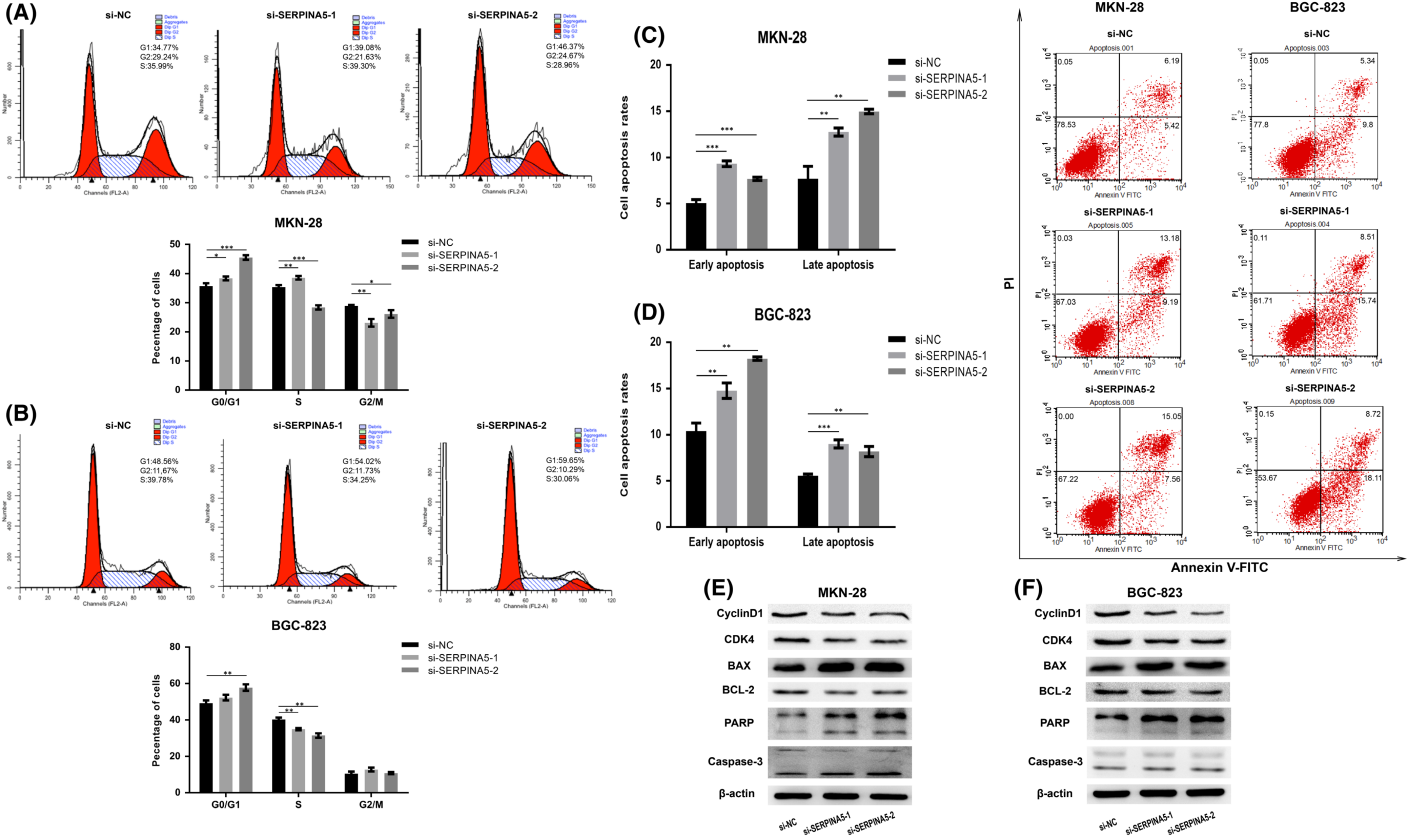
SERPINA5 knockdown induces G0/G1 phase arrest and promotes apoptosis in GC cell lines. Cell cycle distribution of si‐CON and si‐SERPINA5 transfected (A) MKN‐28 and (B) BGC‐823 cells as determined by flow cytometry. Cell apoptosis detection of si‐CON and si‐SERPINA5 transfected (C) MKN‐28 and (D) BGC‐823 cells as determined by flow cytometry. Western blot analysis showing the expression of cell apoptosis and cycle‐related proteins in si‐CON or si‐SERPINA5 transfected (E) MKN‐28 and (F) BGC‐823 cells. *p < 0.05, **p < 0.01, ***p < 0.001

As a means of additionally exploring the mechanisms whereby SERPINA5 drove the proliferation of GC cells, flow cytometry was used to determine cell cycle distribution among different groups. As shown in Figure 3A,B, knocking down SERPINA5 in MKN‐28 and BGC‐823 cells resulted in a considerable elevation in the proportion of cells in G0/G1‐phase. To determine whether apoptosis was also involved in the cell proliferative defect induced by SERPINA5 knockdown, we performed flow cytometry analysis. As showed in Figure 3C,D, there were a significantly elevated apoptosis rates caused by SERPINA5 knockdown in MKN‐28 and BGC‐823 cells. Moreover, the dramatically increased Bax、PARP together with cleaved caspase‐3 and decreased BCL2, CDK‐4, and Cyclin D1 proteins expression levels induced by SERPINA5 knockdown were also observed (Figure 3E,F). Together with our prior results, these findings confirmed that SERPINA5 plays an essential role in the progression of GC in promoting the proliferation ability of human GC cells.

### 3.4 SERPINA5 regulates PI3K/AKT/mTOR signalling pathway through inhibiting CBL

**FIGURE 4:**
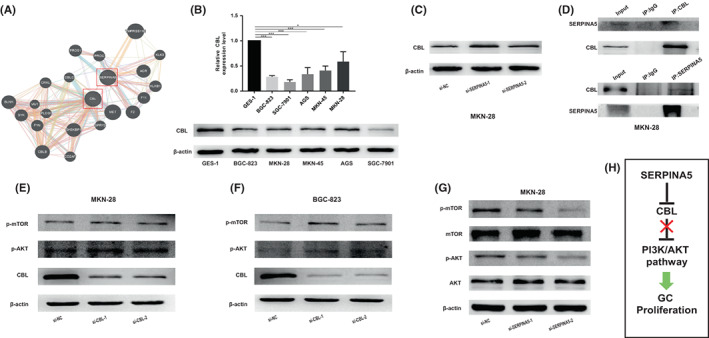
SERPINA5 regulates PI3K/AKT/mTOR signalling pathway through inhibiting CBL. (A) The interaction between SERPINA5 and CBL was showed by GeneMANIA online software. (B) The decreased expression of CBL in GC cell lines both in mRNA and protein levels. (C) The upregulated expression of CBL after silencing SERPINA5 in MKN‐28 cell lines (D) CO‐IP was performed to confirm the interaction between SERPINA5 and CBL in MKN‐28 cell lines. Western blot analysis showing that CBL negatively regulates PI3K/AKT signalling pathway in (E) MKN‐28 and (F) BGC‐823 cells. (G) Knockdown of SERPINA5 can inhibit the PI3K/AKT pathway. (H) The novel mechanism by which high expressed SERPINA5 induces GC progression. *p < 0.05, **p < 0.01, ***p < 0.001

After confirming that SERPINA5 is involved in maintaining the tumorigenic properties of GC cells, the online HitPredict and GeneMANIA software were used to analyse the proteins that may interact with SERPINA5. The results are illustrated in the diagram in Figure 4A by GeneMANIA software and Figure S2 by HitPredict software. Among these proteins, we firstly focused on CBL, an E3 ubiquitin ligase of several tyrosine kinase receptors. CBL exists in a variety of cancers and is mostly considered to be a tumour suppressor gene. It plays a negative regulatory role in many signal transduction pathways and is the gatekeeper to prevent the development of cancer.^28^ Thus, we set out to explore the relationship between CBL and SERPINA5. We firstly detected the low expression of CBL in GC cell lines (BGC‐823, MKN‐28, MKN‐45, AGS and SGC‐7901) compared with GES‐1 in mRNA and protein expression levels (Figure 4B), implying the potential role in tumour inhibition. In addition, we accessed the expression of CBL and showed upregulated after knocking down SERPINA5 in MKN‐28 cell lines (Figure 4C), suggesting that SERPINA5 negatively regulates CBL. Moreover, Co‐IP was performed using antibodies against CBL and SERPINA5, respectively, to further confirm their interaction. As we expected, they indeed bind to each other. (Figure 4D). Collectively, these results indicated that SERPINA5 interacts with CBL and they are negatively correlated. We next further investigated the potential mechanisms underlying the role of SERPINA5 in GC progression. Given that CBL can bind to the P85 subunit of PI3K to promote its ubiquitination, thereby negatively regulating the PI3K/AKT pathway,^29^ which plays a crucial role in proliferation and metastasis of numerous types of cancer, including GC, the modulation of SERPINA5 on the PI3K/AKT/mTOR signalling pathway was thus taken into consideration. First, we proceeded to validate the effects of CBL on PI3K/AKT/mTOR signal by detecting the phosphorylation profiles of AKT (Ser473) and mTOR in MKN‐28 and BGC‐823 cell lines. The results showed that phosphorylated AKT and mTOR were significantly increased when CBL was knocked down (Figure 4E,F), indicating that CBL regulates the PI3K/AKT/mTOR pathway negatively. Then, to address whether PI3K/AKT/mTOR pathway is implicated in SERPINA5‐induced promotion of cell proliferation, the phosphorylation and total expression levels of AKT (Ser 473) and mTOR were measured by Western blot analysis in MKN‐28 cell lines. As is shown in Figure 4G, knockdown of SERPINA5 dramatically inhibited the phosphorylated AKT and mTOR. In summary, our findings suggested that SERPINA5 can regulate the PI3K/AKT/mTOR signalling pathway by inhibiting CBL to promote GC development (Figure 4H).

## 4 DISCUSSION

GC remains one of the most common malignancies, with a high incidence and mortality rate, especially among Asin.^30^ Molecules involved in disease progression are putative prognostic and therapeutic biomarkers.^31^ Hence, discovering successful treatment targets requires a thorough understanding of the events that drive GC growth and development. In our research, we suggested that SERPINA5 functions as a candidate oncogene for GC proliferation and provided a potential novel target for GC treatment.

This study showed that SERPINA5 has a high expression in GC, which is inconsistent with previous reports of SERPINA5 in other tumours.^12^, ^13^, ^32^ However, since SERPINA5 has only been reported in a few tumour types, and the heterogeneity among different tumour types, the previous results are not representative of the expression of SERPINA5 in all tumours. Bioinformatics analysis can help researchers find potential clues from known public dataset.^33^ This study demonstrated that SERPINA5 is correlated with the poor prognosis of patients with GC according to the GEPIA dataset, confirming it may play a certain role in GC. Furthermore, we have been tracking the survival rate of collected GC tissue‐derived patients to further elucidate the prognostic role of SERPINA5. Pervious study demonstrated that loss of the expression of SERPINA5 is correlated with high‐grade tumours on prostate cancer.^13^ In addition, the increased SERPINA5 protein expression is correlated with a prolonging in the survival time of patients with primary breast cancer.^34^ However, it has also reported that upregulation of SERPINA3 is significantly associated with glioma progression and poor patient survival.^35^ Then, our series of experiments indicated that SERPINA5 promotes GC cells proliferation ability by inhibiting cell apoptosis and promoting cell cycle progression, which is completely different from that reported for SERPINA5 in other tumours. Due to the function of uPA in tumour cell metastasis and uPA is inhibited by SERPINA5,^36^, ^37^ SERPINA5 should be closely related to tumour metastasis, and there are indeed some studies that substantiate this point. In breast cancer, overexpression of SERPINA5 inhibits tumour metastasis potential and lowly expressed SERPINA5 inhibits cell migration in hepatocellular carcinoma.^11^, ^18^ In this point, previous study suggested SERPINA5 regulates tumour invasion by inhibiting urokinase‐type plasminogen activator, but regulation tumour growth by SERPINA5 is not dependent on its protease inhibitory activity.^11^ In addition, another study showed that SERPINA5 inhibits tumour growth and promotes tumour metastasis in B16 melanoma.^19^ Taken together, this is the first report demonstrating that SERPINA5 functions as an oncogene in GC, and unlike previous studies, SERPINA5 is not related to cell migration in GC.

To learn more about the underlying mechanism, we tried to predict the potential interactors of SERPINA5, focusing initially on CBL, an E3 ubiquitin ligase of several tyrosine kinase receptors that functions as a suppressor gene in many cancers. We found that they can bind to each other and negatively correlated. CBL is the gatekeeper to prevent the development of cancer and negatively regulate the PI3K/AKT pathway by binding to the P85 subunit of PI3K to promote its ubiquitination.^29^ The PI3K signalling pathway, regulating the cell proliferation and differentiation, is frequently observed to be deregulated in tumours.^38^ Numerous studies have suggested the constitutively activation of PI3K/AKT pathway in GC and the essential role in promoting tumorigenesis.^39^, ^40^, ^41^ As a result, we next explored whether the PI3K/AKT pathway is involved in SERPINA5‐induced GC cell carcinogenesis. We confirmed that CBL inhibits the PI3K/AKT pathway, whereas SERPINA5 silencing can reduce the expression of phosphorylation of p‐AKT at serine 473 and P‐mTOR at Serine 2448. In addition, as a mediator, AKT plays an essential role in the escape of apoptosis. Previous study has demonstrated that AKT can prevent the translocation of Bax from the cytoplasm to the mitochondria, revealing a new mechanism for AKT signalling.^42^ Herein, we also showed that knockdown of SERPINA5 promotes GC cell apoptosis and resulted in decreased p‐AKT expression and increased Bax expression. Therefore, we suggested that SERPINA5 can promote GC carcinogenesis via PI3K/AKT signalling pathway by inhibiting CBL. Although there is currently no published evidence for the interaction of SERPINA5 and CBL, the prediction server database indeed provides clues that these two gene products are linked like physical interaction. We supposed that there may be multiple molecular interactions besides SERPINA5 and CBL. It is very likely that there has an intermediate molecule, which not only has upregulated expression lead by the inactivation of serine proteases due to the presence of SERPINA5, but also negatively regulates the gene expression of CBL, thereby affecting the subsequent signal transduction, these requires further research. This is the first study providing evidence that PI3K/AKT pathway was identified as an important pathway involved in the function of SERPINA5 in regulating GC progression. Studying the interaction mechanism of SERPINA5 and CBL is the main content of future work. Furthermore, in vivo experiments and more in‐depth mechanism research are necessary to confirm the possibility of SERPINA5 in tumour therapy.

In conclusion, this study showed the increased SERPINA5 expression in GC and indicated that SERPINA5 functions as an oncogene to promote proliferation in GC cells via PI3K/AKT pathway, which may provide a therapeutic target for the future treatment.

## AUTHOR CONTRIBUTIONS

**Meiyang Fan:** Data curation (equal); formal analysis (equal); validation (equal); visualization (equal); writing – original draft (equal). **Xiaofan Xiong:** Data curation (equal); formal analysis (equal); investigation (equal); validation (equal). **Lin Han:** Data curation (equal); investigation (equal); methodology (equal). **Lingyu Zhang:** Data curation (equal); validation (equal). **Shanfeng Gao:** Data curation (equal); resources (equal). **Liying Liu:** Formal analysis (equal); software (equal). **Xiaofei Wang:** Formal analysis (equal); software (equal). **Chen Huang:** Validation (equal); writing – review and editing (equal). **Dongdong Tong:** Validation (equal); writing – review and editing (equal). **Juan Yang:** Methodology (equal); validation (equal); writing – review and editing (equal). **Ling‐yu Zhao:** Methodology (equal); validation (equal). **Yuan Shao:** Methodology (equal); supervision (equal); validation (equal).

## CONFLICT OF INTEREST

The authors declare that they have no competing interests.

## Supporting information


Figures S1‐S2

## Data Availability

The data that support the findings of this study are available from the corresponding author upon reasonable request." cd_value_code="text
