# Supplementary material for: SERPINA5 promotes tumour cell proliferation by modulating the PI3K/AKT/mTOR signalling pathway in gastric cancer
Source: J Cell Mol Med. 2022 Aug 24;26(18):4837–46. doi: 10.1111/jcmm.17514 (PMC9465189; doi:10.1111/jcmm.17514)
Supplement: Supplementary file 1 — Figures S1‐S2 [file JCMM-26-4837-s001.pdf]

SERPINA5 promotes tumor cell proliferation by modulating the PI3K/ATK/mTOR signaling pathway in gastric cancer

Meiyang Fan[1,2,3], Xiaofan Xiong[4], Lin Han[3], Lingyu Zhang[2], Shanfeng Gao[2], Liying Liu[3], Xiaofei Wang[3], Chen Huang[3], Dongdong Tong[2], Juan Yang[2,3,*], Lingyu Zhao[2,3,*], Yuan Shao[1,*]

[1]Department of Otolaryngology & Head Neck, the First Affiliated Hospital of Xi'an Jiaotong University, Xi'an, 710061, P. R. China.

[2]Department of Cell Biology and Genetics, School of Basic Medical Sciences, Xi'an Jiaotong University Health Science Center, Xi'an 710061, P. R. China.

[3]Key Laboratory of Environment and Genes Related to Diseases (Xi'an Jiaotong University), Ministry of Education of China, Xi'an 710061, P. R. China.

[4]Department of Tumor and Immunology in precision medicine institute, Western China Science and Technology Innovation Port, Xi'an, 710004, P. R. China.

*Correspondence: Yuan Shao (Email: Shaxiaoying1976@163.com)

*Co-correspondence: Lingyu Zhao (Email: zhaolingyu@xjtu.edu.cn)

*Co-correspondence: Juan Yang (Email: yangjuan0112@xjtu.edu.cn)

Figure S1

SERPINA5 has no impact on the migration of GC cells.

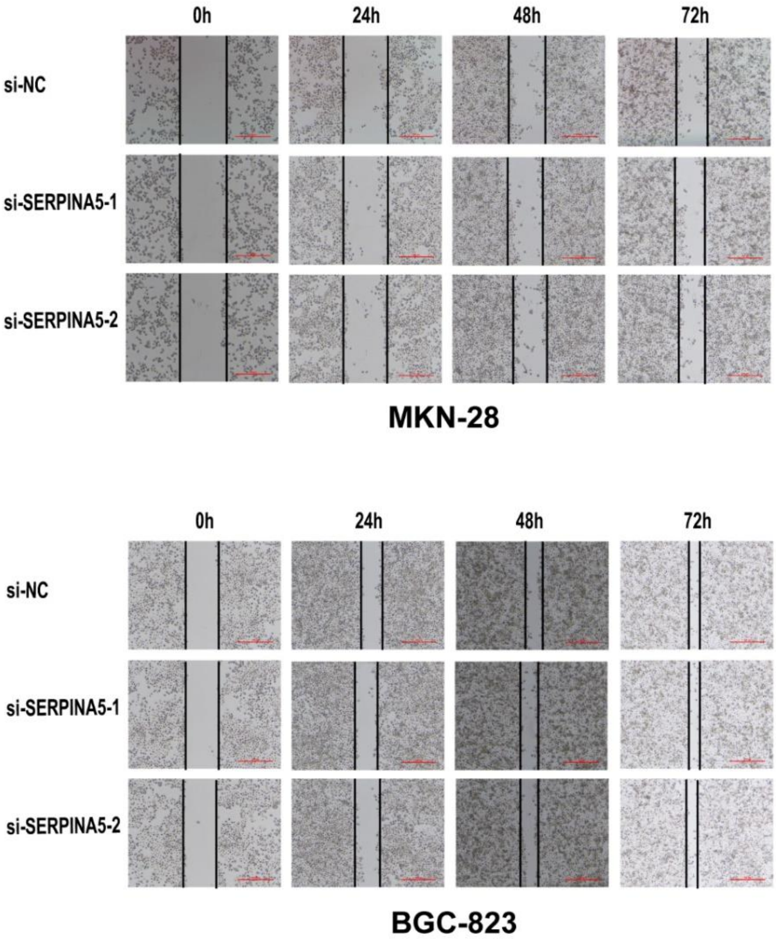

Figure S2

There has been the interaction between SERPINA5 and CBL by HitPredict.

Interaction	Interactor	Experiments	Homologs	Category	Interaction Score	Confidence
204430	KLK3	2	15	Small-scale	0.811	High
169491	UROK	2	20	Small-scale	0.778	High
190955	PROC	2	20	Small-scale	0.755	High
204439	HGFA	1	20	High-throughput	0.691	High
169322	THRB	1	20	Small-scale	0.647	High
169515	TPA	1	20	Small-scale	0.647	High
189755	FA11	1	20	Small-scale	0.647	High
189761	KLKB1	1	13	Small-scale	0.647	High
204432	ACRO	1	8	Small-scale	0.647	High
204434	KLK2	1	14	Small-scale	0.618	High
204441	EPS8	2	1	High-throughput	0.576	High
204431	FURIN	1	1	High-throughput	0.536	High
204436	CALX	1	1	High-throughput	0.536	High
204443	TGO1	1	6	High-throughput	0.536	High
204435	CBL	2	1	High-throughput	0.526	High
204445	CSN6	1	3	High-throughput	0.494	High
204446	PIAS4	1	1	High-throughput	0.494	High
204449	ZN363	1	1	High-throughput	0.494	High
88813	ZZEF1	1	1	High-throughput	0.489	High
204444	HECD3	1	1	High-throughput	0.489	High
204450	WWOX	1	1	High-throughput	0.489	High
204452	FBX2	1	2	High-throughput	0.489	High
204437	BAP31	1	1	High-throughput	0.479	High
204451	BAP29	1	1	High-throughput	0.479	High
204438	RIDA	1	0	High-throughput	0.460	High
72887	PGRC2	1	0	High-throughput	0.458	High
204442	MSMO1	1	0	High-throughput	0.458	High
204448	MIA2	1	7	High-throughput	0.445	High
123260	SC22B	1	1	High-throughput	0.437	High
204440	EP300	1	1	High-throughput	0.417	High
204447	ESIP1	1	0	High-throughput	0.279	Low
204433	H15	1	0	High-throughput	0.249	Low
